# Comparison of Neuromuscular Control Characteristics in Forehand Stroke Between International- and National-Level Squash Players: An sEMG-Based Analysis of Muscle Synergy and Intermuscular Coherence

**DOI:** 10.3390/s26123840

**Published:** 2026-06-17

**Authors:** Hao Zhang, Bingnan Wang, Jiao Tong, Yanan Shen

**Affiliations:** Department of P.E., Central South University, Changsha 410083, China; csu19971423619@163.com (H.Z.); tongjiao1985@hotmail.com (J.T.); shenyanan2016@163.com (Y.S.)

**Keywords:** surface electromyography, wearable sensors, biosignal sensing, muscle synergy, intermuscular coherence, neuromuscular control, squash, sports biomechanics, athlete monitoring

## Abstract

Objective: This study aimed to compare the neuromuscular control characteristics of international- and national-level squash players during forehand strokes using a multichannel surface electromyography (sEMG)-based sensing framework. By integrating wearable biosignal acquisition with muscle synergy and intermuscular coherence analyses, this study sought to identify sensor-derived markers of performance-related neuromuscular control and to provide evidence for sensor-informed squash training and athlete monitoring. Methods: Participants performed standardized forehand strokes, during which multichannel sEMG signals were synchronously collected from major upper-limb, lower-limb, and trunk muscles. The recorded sensor signals were preprocessed and analyzed using non-negative matrix factorization to extract muscle synergies, including the number of synergies, muscle weightings, and synergy activation durations. In addition, time–frequency intermuscular coherence analysis was performed on the sEMG sensor data to quantify coherence differences in the α, β, and γ frequency bands between upper-limb–trunk and lower-limb–trunk muscle pairs. Results: No significant difference was found between the two groups in the number of muscle synergies, with both groups clustering into four synergy modules. However, the sEMG sensor-based analysis revealed clear between-group differences in synergy structure and coordination patterns. International-level players showed higher muscle weightings in major proximal muscles, including the deltoid, pectoralis major, erector spinae, and gluteus maximus, and lower weightings in relatively smaller or more distal muscles such as the biceps brachii and lateral gastrocnemius. In terms of synergy timing, international-level players exhibited significantly shorter activation durations in SYN1 and SYN2, but a significantly longer activation duration in SYN3, than national-level players. For intermuscular coherence, international-level players showed significantly lower coherence in the α, β, and γ bands for multiple upper-limb–trunk and lower-limb–trunk muscle pairs. Conclusions: A multichannel sEMG sensing approach was effective in detecting performance-level differences in neuromuscular control during the squash forehand stroke. International-level players exhibited more efficient and refined neuromuscular coordination, characterized by optimized proximal muscle recruitment, more task-specific synergy timing, and reduced intermuscular coherence across selected muscle pairs. These findings highlight the value of wearable EMG sensors and sensor-based neuromuscular feature extraction for quantitative athlete assessment, movement monitoring, and the development of sensor-guided training strategies in squash.

## 1. Introduction

Squash is a high-intensity intermittent racket sport characterized by repeated accelerations, decelerations, rapid changes of direction, and high-frequency strokes [1,2]. Among the major stroke techniques, the forehand stroke is a key offensive action because it requires rapid force generation, precise racket control, and coordinated transfer of mechanical energy from the lower limbs and trunk to the upper limb and racket [3,4]. Therefore, understanding how skilled players organize whole-body neuromuscular coordination during the forehand stroke is important for explaining performance-level differences and for developing more targeted training strategies.

Previous studies on squash performance have mainly focused on physiological demands, match-related fatigue, and stroke kinematics. Squash match play has been shown to impose substantial cardiovascular and neuromuscular demands [3], and squash-specific exercise can alter neuromuscular function, indicating that the sport places considerable stress on the neuromuscular system [4]. With respect to stroke technique, Williams et al. (2020) and Pan et al. (2024) reported that higher-skilled squash players exhibited greater joint range of motion and higher swing velocity during forehand strokes, whereas lower-skilled players showed slower racket speed and a more open racket face at ball impact [5,6]. These findings demonstrate that skill level is associated with clear biomechanical differences during the squash forehand. However, kinematic variables primarily describe the external manifestation of movement and do not directly explain how the central nervous system organizes multi-muscle activation to produce these performance differences.

Surface electromyography (sEMG) provides a direct method for examining muscle activation during sport-specific movements. Nevertheless, existing EMG-based evidence in squash remains limited, and previous work has mainly described muscle activation levels rather than the organization of neuromuscular control during the forehand stroke. In particular, it remains unclear whether international-level and national-level squash players differ in the modular organization of muscle activation, the temporal recruitment of muscle modules, or the neural coupling among muscles during the forehand stroke. This gap is important because the squash forehand is not an isolated upper-limb action; rather, it depends on coordinated interaction among the lower limbs, trunk, shoulder complex, and forearm. Therefore, methods capable of quantifying both multi-muscle coordination structure and intermuscular neural coupling are needed to move beyond conventional kinematic or single-muscle EMG comparisons.

Muscle synergy analysis provides a useful framework for addressing this issue. The muscle synergy concept proposes that the central nervous system may simplify the control of complex movements by activating a limited number of coordinated muscle modules, rather than controlling each muscle independently [7,8]. In this framework, each synergy represents a relatively invariant pattern of muscle weighting across multiple muscles, whereas the corresponding activation coefficient describes how strongly and when that module is recruited during movement [7,9]. By applying non-negative matrix factorization to multichannel sEMG signals, muscle synergy analysis can identify the number of synergies, the contribution of individual muscles within each synergy, and the temporal activation profile of each module [10].

The methodological value of muscle synergy analysis has been demonstrated in both basic motor control research and clinical rehabilitation. Foundational studies have shown that a small set of muscle synergies can account for complex activation patterns during posture, locomotion, and natural motor behaviors, supporting the idea that modular organization is a general strategy of neuromuscular control [11]. In clinical populations, muscle synergy analysis has been used to characterize altered motor coordination after neurological injury, especially stroke [9,12,13]. For example, muscle synergy patterns have been proposed as physiological markers of motor cortical damage after stroke, and the merging of healthy motor modules has been associated with reduced locomotor performance and decreased muscle coordination complexity in post-stroke gait. These clinical applications show that muscle synergy analysis can provide information about underlying neural strategies that cannot be obtained from clinical scales, kinematics, or single-muscle activation amplitudes alone.

The relevance of muscle synergy analysis is not limited to clinical populations. In sport and other complex motor tasks, the framework can be used to examine how expertise, training, and task constraints influence multi-muscle coordination [14,15]. Importantly, skill-related differences may not necessarily appear as a change in the total number of synergies. Instead, they may be reflected in the composition of muscle weightings, the relative contribution of proximal and distal muscles, and the timing or duration of synergy activation. For the squash forehand, this distinction is particularly relevant because superior performance may depend less on recruiting more muscle modules and more on optimizing how existing modules are organized and timed across the kinetic chain [16,17]. Thus, muscle synergy analysis is well suited to determining whether international-level players use a more refined coordination strategy than national-level players during the forehand stroke.

Intermuscular coherence (IMC) provides complementary information by quantifying frequency-domain coupling between EMG signals from different muscles [18]. Whereas muscle synergy analysis describes the modular structure and temporal recruitment of muscle activation, IMC reflects the extent to which different muscles share common oscillatory neural input [19,20]. Coherence in the α, β, and γ frequency bands has been associated with different components of neuromuscular control, and β-band coherence is commonly interpreted as an indicator of common corticospinal drive [21,22]. Laine and Valero-Cuevas further suggested that IMC can reflect functional coordination among coactivated muscles [23]. Therefore, combining muscle synergy and IMC analyses allows for the squash forehand to be examined from two complementary perspectives: the organization of multi-muscle activation modules and the neural coupling underlying intermuscular coordination.

Despite existing evidence of skill-level differences in squash forehand kinematics [5], it remains unknown whether these biomechanical differences are accompanied by distinct neuromuscular control strategies. Specifically, no study has systematically compared international-level and national-level squash players during the forehand stroke using both muscle synergy and IMC analyses. Addressing this gap may clarify whether superior squash performance is associated with differences in synergy number, muscle weighting distribution, activation timing, and intermuscular neural coupling. Therefore, this study aimed to compare neuromuscular control characteristics during the forehand stroke between international-level and national-level squash players using multichannel sEMG-based muscle synergy and IMC analyses. We hypothesized that international-level players would show more refined neuromuscular organization, characterized by optimized muscle weighting patterns, more task-specific synergy activation timing, and altered intermuscular coherence compared with national-level players.

## 2. Methods

### 2.1. Participants

A total of 24 male squash players were recruited in this study, including 10 athletes in the international group (INT) and 14 athletes in the national group (NAT). Players in the NAT group were elite members of the Chinese National Squash Team, including medalists who finished in the top three at the National Championships (mean age: 22.50 ± 4.11 years; height: 185.0 ± 4.0 cm; body mass: 74.7 ± 7.4 kg). Players in the INT group were ranked within the top 50 of the Professional Squash Association (PSA) and had achieved top-three finishes in world-class competitions (mean age: 27.60 ± 2.80 years; height: 179.0 ± 6.0 cm; body mass: 72.5 ± 7.7 kg). All participants were right-handed racket holders, reported no history of major musculoskeletal injury, and provided written informed consent prior to testing. Participant characteristics are summarized in Table 1. Written informed consent was obtained from all participants prior to enrollment, and the study was approved by the institutional ethics committee.

### 2.2. Experimental Procedures

#### 2.2.1. Instrumentation

The sEMG system (Noraxon, Scottsdale, AZ, USA) consisted of a 16-channel wireless acquisition unit with a sampling frequency of 2000 Hz, an input bandwidth of 10–500 Hz, and a common-mode rejection ratio (CMRR) > 110 dB. The 10–500 Hz range refers to the manufacturer-specified hardware acquisition bandwidth and was used to describe the recording characteristics of the device, rather than the parameters of the offline digital filtering. No additional filtering or signal preprocessing was applied in the Noraxon acquisition software 2.1.10, except for real-time visualization and data export. After export, the raw sEMG data were processed offline using custom scripts. All electrode placement and skin preparation procedures complied with the SENIAM recommendations [24].

Kinematic data were captured using a high-speed camera-based motion capture system operating at 200 Hz. The capture volume was calibrated using a 12-point three-dimensional calibration frame (1 × 1 × 0.8 m), yielding a reprojection error < 0.3 mm. Temporal synchronization between the sEMG and motion-capture systems was achieved using a shared external hardware trigger. Specifically, a transistor–transistor logic (TTL) pulse generated by the trigger unit was simultaneously sent to the digital input channel of the Noraxon sEMG acquisition system and to the synchronization input of the high-speed motion-capture system before each recording trial. The rising edge of this pulse was recorded as time zero in both systems and was used as the common temporal reference for subsequent signal alignment. During offline processing, the trigger markers recorded in the sEMG and kinematic data streams were identified, and all EMG and motion capture signals were aligned to the same trigger onset before extracting the analysis window from 200 ms before ball contact to 300 ms after ball contact. This procedure ensured that muscle activation and kinematic events were analyzed within the same movement cycle. Standardized equipment was used throughout the experiment, including a regulation squash racket, competition balls, and a ball-feeding machine, to ensure consistent testing conditions.

#### 2.2.2. Experimental Protocol

Maximal voluntary contraction (MVC) testing: Prior to the main experiment, MVC tests were performed for all target muscles to provide reference values for EMG amplitude normalization. All MVC trials were conducted under standardized isometric conditions rather than dynamic conditions, because isometric MVC testing provides a controlled and reproducible reference for EMG normalization during subsequent sport-specific movements [25,26]. For each target muscle, participants performed three maximal 5 s contractions against manual or fixed resistance, with a 60 s rest interval between trials to minimize fatigue. Strong verbal encouragement was provided during each trial. The highest EMG amplitude obtained across the three trials for each muscle was used as the normalization reference for that muscle.

The MVC positions were selected according to the primary anatomical function of each muscle. For the tibialis anterior, participants performed resisted ankle dorsiflexion in a seated position. For the medial and lateral gastrocnemius, participants performed resisted ankle plantar flexion with the knee extended. For the vastus medialis and vastus lateralis, participants performed resisted knee extension in a seated position. For the biceps femoris, participants performed resisted knee flexion. For the gluteus maximus, participants performed resisted hip extension in a prone position. For the erector spinae, participants performed resisted trunk extension. For the latissimus dorsi, participants performed resisted shoulder extension and adduction. For the trapezius, participants performed resisted shoulder elevation. For the pectoralis major, participants performed resisted horizontal shoulder adduction. For the biceps brachii, participants performed resisted elbow flexion with the forearm supinated. For the deltoid, participants performed resisted shoulder abduction. For the brachioradialis, participants performed resisted elbow flexion with the forearm in a neutral position. These standardized MVC procedures were used only to obtain normalization references and were not intended to reproduce the dynamic forehand stroke movement.

Forehand stroke task: Testing was conducted on a standard squash court. A ball-feeding machine delivered balls at a fixed frequency. Participants performed 20 consecutive forehand strokes at a match-like pace, aiming at a predefined target area (50 cm × 50 cm). sEMG and kinematic signals were recorded simultaneously. Invalid trials were repeated.

Synchronization and analysis window: An external trigger signal was used to initiate synchronous recording in both systems. The sampling window was defined from 200 ms before ball contact to 300 ms after ball contact.

Group allocation and testing conditions: The international and national groups followed identical procedures, equipment settings, and environmental conditions to ensure comparability of the measurements.

### 2.3. sEMG Acquisition

sEMG signals were recorded from 14 target muscles using the Noraxon wireless system. The measured muscles included the lower-limb muscles: tibialis anterior (TA), medial gastrocnemius (GM), lateral gastrocnemius (GL), vastus medialis (VM), vastus lateralis (VL), biceps femoris (BF), and gluteus maximus (GLM); as well as trunk and upper-limb muscles: erector spinae (ES), latissimus dorsi (LD), trapezius (TRAP), pectoralis major (PM), biceps brachii (BB), deltoid (DEL), and brachioradialis (BRD).

Before electrode placement, the skin was cleaned with 75% alcohol to remove surface oils and dead skin, thereby reducing skin–electrode impedance and improving signal quality. Electrode placement strictly followed SENIAM guidelines, and electrodes were secured with medical tape to minimize displacement during large-amplitude movements.

For analysis purposes, the forehand stroke was divided into four characteristic phases: stance phase, forehand swing (backswing) phase, swing and contact point phase, and forehand follow-through phase [21].

sEMG signals from the specified muscles were recorded throughout the forehand stroke and subsequently analyzed. Intermuscular coherence analysis was conducted to quantify functional coupling between muscles, and muscle synergies were extracted using non-negative matrix factorization (NMF) to investigate between-group differences in intermuscular coordination and synergy organization between international-level and national-level players.

### 2.4. Computation of Muscle Synergies and Intermuscular Coherence

Muscle synergy extraction and intermuscular coherence analyses were performed using a combined R and Python workflow. Muscle synergy extraction, including non-negative matrix factorization, VAF calculation, and clustering of synergy vectors, was performed in R version 4.2.0 using the musclesyneRgies package version 1.2.5. Offline sEMG preprocessing, time normalization, and time–frequency intermuscular coherence analysis were performed in Python version 3.9 using custom scripts. The main Python packages used were NumPy, SciPy, Pandas, and Matplotlib 3.1. Specifically, SciPy was used for digital filtering, short-time Fourier transform, spectral estimation, and convolution-based smoothing, while NumPy and Pandas were used for numerical computation and data organization. The overall workflow is described below.

#### 2.4.1. EMG Signal Processing

Raw sEMG data were first exported from the Noraxon acquisition software without additional offline filtering in the acquisition software. All subsequent preprocessing steps were performed offline in Python version 3.9 using custom scripts based on NumPy, SciPy, and Pandas. For muscle synergy analysis, the raw sEMG signals were first band-pass filtered using a fourth-order Butterworth filter between 20 and 400 Hz to attenuate movement artifacts and high-frequency noise. The filtered signals were then full-wave rectified and smoothed using a fourth-order low-pass Butterworth filter with a cutoff frequency of 20 Hz to obtain the linear envelope. The resulting EMG envelopes were normalized to each participant’s maximal voluntary contraction value for the corresponding muscle. To minimize the influence of inter-trial differences in movement duration, each processed EMG signal was time-normalized by interpolation to 100 data points over the analysis window from 200 ms before ball contact to 300 ms after ball contact. The time-normalized and MVC-normalized EMG envelopes were then imported into R version 4.2.0 for muscle synergy extraction using the musclesyneRgies package version 1.2.5.

For intermuscular coherence analysis, preprocessing was performed separately in Python. The raw sEMG signals were band-pass filtered between 20 and 400 Hz using the same fourth-order Butterworth filter. The filtered signals were then full-wave rectified before time–frequency coherence estimation. Rectification was applied because previous EMG coherence studies have suggested that rectified EMG may enhance the detection of common neural input to muscles by emphasizing the modulation of motor-unit activity. However, because EMG preprocessing choices can influence coherence estimates, the same preprocessing procedure was applied consistently to all participants and both groups. In addition, the coherence results were interpreted as relative between-group differences rather than as direct measures of absolute neural drive.

For the time–frequency coherence procedure, rectified EMG signals were segmented using a Hamming window of 200 samples with 75% overlap. Cross-spectral and auto-spectral densities were estimated using a short-time Fourier transform. A two-dimensional convolution-based smoothing procedure was then applied to the spectral estimates before coherence calculation. Coherence was quantified within the α (8–15 Hz), β (15–30 Hz), and γ (30–50 Hz) frequency bands. These preprocessing and coherence-analysis steps were implemented entirely in Python using custom scripts.

#### 2.4.2. Muscle Synergy Extraction

Muscle synergies were extracted from the processed EMG data using NMF. The EMG data matrix D(t) was decomposed into a set of time-invariant synergy vectors $W_{i}$ (muscle weightings) and time-varying activation coefficients $C_{i}(t)$, such that:

$$D(t)=\sum_{i=1}^{N_{syn}}C_{i}(t)W_{i}$$

where $N_{syn}$ denotes the number of synergies. To determine the optimal $N_{syn}$, NMF was performed iteratively with the number of synergies ranging from 1 to 14. The minimum number of synergies required to explain 90% of the variance accounted for (VAF) in EMG reconstruction was selected. VAF was computed as:

$$VAF=1-\frac{SSE}{SST}$$

where SST is the total sum of squares and SSE is the sum of squared reconstruction errors. NMF was initialized with random values uniformly distributed between 0 and the maximum EMG amplitude. Iterations were terminated once VAF > 0.90, and the solution yielding the highest VAF was retained for subsequent analyses.

#### 2.4.3. Clustering and Matching of Synergy Patterns

K-means clustering (squared Euclidean distance; 1000 repetitions) was applied to identify representative synergy vectors within each group. The optimal number of clusters was determined using the Gap statistic. The cluster number *k* was selected when the following criterion was satisfied:

Gap(k)≥Gap(k+1)−sd(k+1)

where sd(k) represents the standard deviation of Gap values estimated from reference datasets. After clustering, synergies from the other group were assigned to the corresponding reference synergies based on the correlation coefficient of muscle weightings (threshold r ≥ 0.6), enabling between-group comparisons of synergy patterns.

#### 2.4.4. Time–Frequency Intermuscular Coherence

Time–frequency intermuscular coherence was computed in Python version 3.9 using custom scripts based on NumPy and SciPy. Coherence analysis was performed on band-pass filtered and full-wave rectified EMG signals. The use of rectified EMG was selected because rectification has been commonly used in EMG coherence studies to enhance the representation of amplitude modulation related to common neural input across muscles. Nevertheless, because the effects of rectification on coherence estimation remain method-dependent, the same preprocessing pipeline was applied to all participants, trials, muscles, and groups to ensure that the between-group comparisons were not biased by inconsistent signal processing.

The rectified EMG signals were segmented using a Hamming window of 200 samples with 75% overlap. For each window, cross-spectral density between two EMG signals and the corresponding auto-spectral densities were estimated using a short-time Fourier transform. The spectral estimates were then smoothed using a two-dimensional convolution kernel in the time–frequency domain. Time–frequency coherence was computed as:

Cxy(l,f)=|p^xy[l,f]⊗v[t]|2{|p^xx[l,f]|2⊗v[t]}{|p^yy[l,f]|2⊗v[t]}

where p^xy[l,f] denotes the cross-spectrum between EMG signals x and y, p^xx[l,f] and p^yy[l,f] denote the corresponding auto-spectra, ⊗ indicates convolution, and v denotes the smoothing kernel. TFC values were normalized to a range of 0–1, with values closer to 1 indicating stronger coherence. Significant coherence regions were quantified within the α (8–15 Hz), β (15–30 Hz), and γ (30–50 Hz) bands. Group comparisons were conducted for trunk–lower limb and trunk–upper limb muscle pairs.

### 2.5. Statistical Analysis

All statistical analyses were performed using SPSS Statistics version 26.0 (SPSS Inc., Chicago, IL, USA). The statistical analysis was designed to test whether international-level and national-level squash players differed in four main neuromuscular outcomes during the forehand stroke: the number of extracted muscle synergies, the muscle weighting coefficients within each synergy, the activation duration of each synergy, and the time–frequency intermuscular coherence values. These comparisons were intended to determine whether performance-level differences were reflected in the dimensionality of modular control, the distribution of muscle contributions within each module, the temporal recruitment of synergy modules, and the strength of intermuscular neural coupling.

For the number of extracted synergies, a between-group comparison was conducted to examine whether international-level players used a different number of synergy modules than national-level players. For muscle weighting coefficients, between-group comparisons were performed separately for each muscle within each matched synergy to identify whether the two groups differed in the relative contribution of individual muscles to each synergy module. For synergy activation duration, between-group comparisons were performed for each synergy to assess whether the temporal persistence of synergy recruitment differed between groups. For intermuscular coherence, between-group comparisons were conducted for the Az values of selected upper limb–trunk and lower limb–trunk muscle pairs within the α, β, and γ frequency bands to determine whether international-level players exhibited different patterns of intermuscular neural coupling.

Prior to statistical testing, boxplots were used to screen for extreme outliers. Data normality was assessed using the Shapiro–Wilk test. For variables that satisfied the normality assumption, between-group comparisons were conducted using independent-samples *t* tests. For variables that did not satisfy the normality assumption, the Mann–Whitney U test was used.

Because multiple between-group comparisons were performed, the Benjamini–Hochberg false discovery rate (FDR) correction was applied to control for multiple testing. The correction families were defined a priori according to the structure of each outcome measure. For muscle weighting coefficients, FDR correction was applied separately within each synergy across the 14 muscle comparisons; therefore, SYN1, SYN2, SYN3, and SYN4 were treated as four separate correction families. For synergy activation duration, FDR correction was applied across the four synergy comparisons. For intermuscular coherence, FDR correction was applied separately for upper limb–trunk and lower limb–trunk muscle pair comparisons within each frequency band. Thus, α-, β-, and γ-band coherence comparisons were corrected within their respective anatomical muscle pair categories. Unless otherwise stated, *p* values reported in the Section 3, Tables 3 and 4, and related figures are FDR-adjusted *p* values. Statistical significance was defined as an FDR-adjusted *p* value < 0.05. All *p* values reported in the Section 3, Tables 3 and 4, and related figures are Benjamini–Hochberg FDR-adjusted *p* values unless otherwise specified. For muscle weighting coefficients, FDR correction was applied separately within each synergy across the 14 muscle comparisons. For synergy activation duration, FDR correction was applied across the four synergy comparisons. For intermuscular coherence, FDR correction was applied separately for upper limb–trunk and lower limb–trunk muscle pair comparisons within each frequency band.

## 3. Results

### 3.1. Comparison of the Total Number of Muscle Synergies Between INT and NAT Players

As shown in Table 2, under the criterion of VAF > 0.90, no significant between-group difference was observed in the number of extracted synergies. Both the INT and NAT groups typically exhibited approximately five synergies.

### 3.2. Between-Group Differences in Muscle Weightings

**Figure 1:**
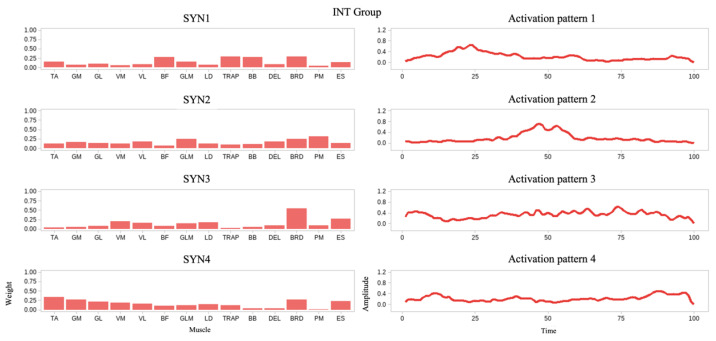
Synergy pattern analysis of SYN1–SYN4 in the international-level group (INT). For each synergy, the left panel shows the muscle weighting coefficients, which indicate the relative contribution of each muscle to the corresponding synergy module, and the right panel shows the temporal activation coefficient of that synergy during the forehand stroke. The *x*-axis of the activation profile represents the time-normalized forehand stroke cycle, and the *y*-axis represents the normalized activation coefficient. A synergy module was considered active when its activation coefficient exceeded 0.30. Muscle abbreviations are as follows: tibialis anterior (TA), medial gastrocnemius (GM), lateral gastrocnemius (GL), vastus medialis (VM), vastus lateralis (VL), biceps femoris (BF), gluteus maximus (GLM), erector spinae (ES), latissimus dorsi (LD), trapezius (TRAP), pectoralis major (PM), biceps brachii (BB), deltoid (DEL), and brachioradialis (BRD).

**Figure 2:**
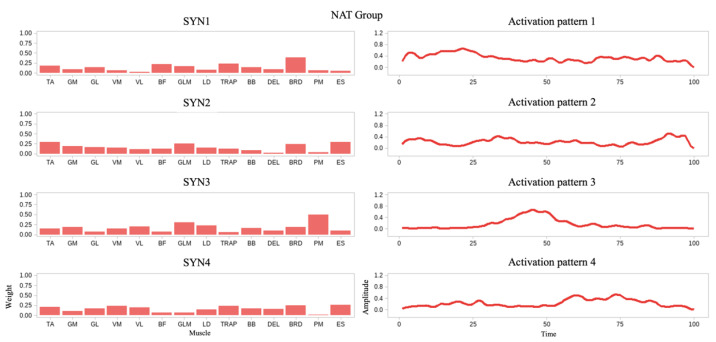
Synergy pattern analysis of SYN1–SYN4 in the national-level group (NAT). For each synergy, the left panel shows the muscle weighting coefficients, which indicate the relative contribution of each muscle to the corresponding synergy module, and the right panel shows the temporal activation coefficient of that synergy during the forehand stroke. The *x*-axis of the activation profile represents the time-normalized forehand stroke cycle, and the *y*-axis represents the normalized activation coefficient. A synergy module was considered active when its activation coefficient exceeded 0.30. Muscle abbreviations are as follows: tibialis anterior (TA), medial gastrocnemius (GM), lateral gastrocnemius (GL), vastus medialis (VM), vastus lateralis (VL), biceps femoris (BF), gluteus maximus (GLM), erector spinae (ES), latissimus dorsi (LD), trapezius (TRAP), pectoralis major (PM), biceps brachii (BB), deltoid (DEL), and brachioradialis (BRD).

**Figure 3:**
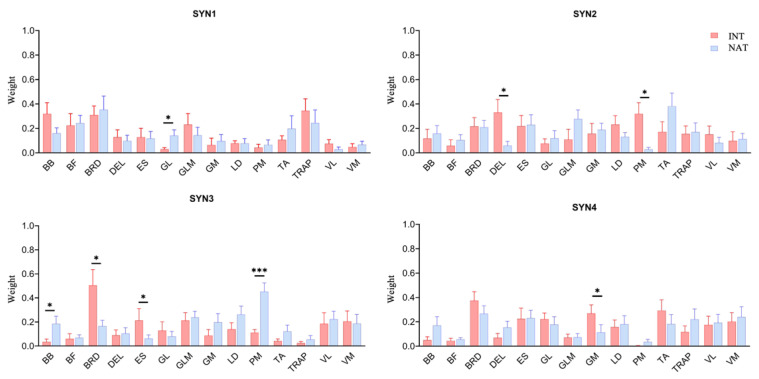
Muscle weightings for SYN1–SYN4 in the INT and NAT groups. *p* values were FDR-adjusted within each synergy across the 14 muscle comparisons. * indicates FDR-adjusted *p* < 0.05; *** indicates FDR-adjusted *p* < 0.001. Significance markers indicate FDR-adjusted *p* values.

Figure 1, Figure 2 and Figure 3 and Table 3 indicate between-group differences in the weighting coefficients of specific muscles within several synergies. Specifically, in SYN1, the weighting of GL was lower in the INT group than in the NAT group (*p* = 0.016). In SYN2, the weightings of DEL and PM were higher in INT than in NAT (both *p* = 0.020). In SYN3, the weighting of BB was lower in INT than in NAT (*p* = 0.038), whereas the weightings of BRD and ES were higher in INT than in NAT (*p* = 0.035 and *p* = 0.047, respectively); additionally, the weighting of PM was lower in INT than in NAT (*p* = 0.0008). In SYN4, the weighting of GM was higher in INT than in NAT (*p* = 0.040).

### 3.3. Comparison of Synergy Activation Duration Between INT and NAT Players

**Figure 4:**
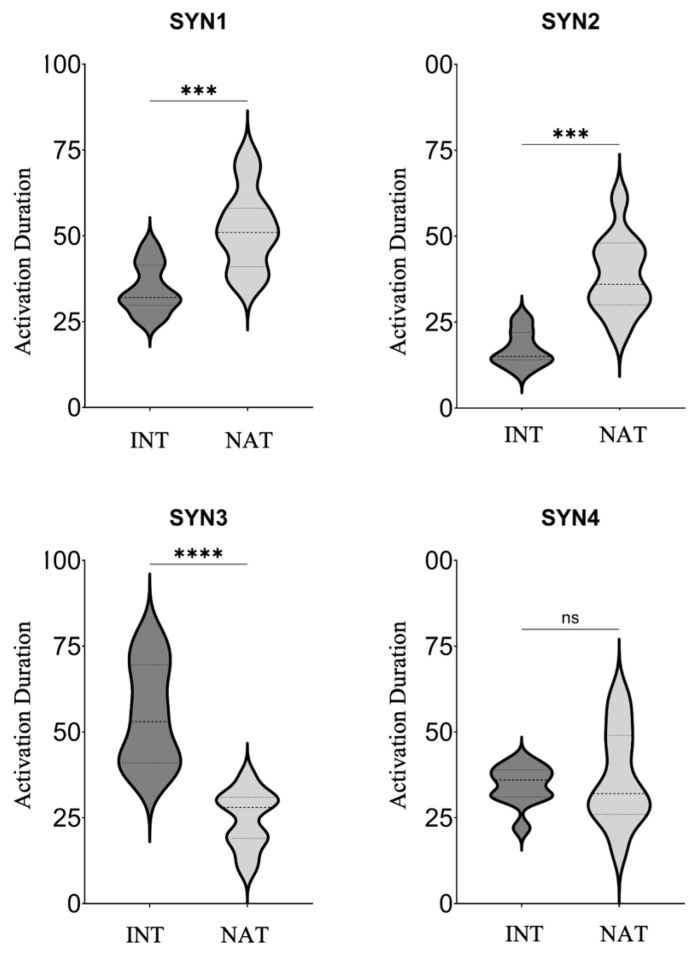
Comparison of synergy activation duration between INT and NAT players. Violin plots show the distribution of activation duration for SYN1–SYN4 in the international-level group (INT) and national-level group (NAT). Activation duration was defined as the period during which the synergy activation coefficient exceeded 0.30. *p* values were FDR-adjusted across the four synergy comparisons. The violin width indicates data density, and the dashed horizontal lines indicate the central tendency. Significance markers indicate between-group differences: ns, not significant; ***, FDR-adjusted *p* < 0.001; ****, FDR-adjusted *p* < 0.0001. Significance markers indicate FDR-adjusted *p* values.

Synergy activation duration was defined as the time interval during which the synergy activation coefficient exceeded 0.30. As shown in Table 4 and Figure 4, the INT group exhibited a significantly shorter activation duration than the NAT group for SYN1 (*p* = 0.0006) and SYN2 (*p* = 0.002). In contrast, the activation duration of SYN3 was significantly longer in INT than in NAT (*p* < 0.0001). No significant between-group difference was observed for SYN4 (Table 4 and Figure 4).

### 3.4. Comparison of Time–Frequency Intermuscular Coherence Between INT and NAT Players

**Figure 5:**
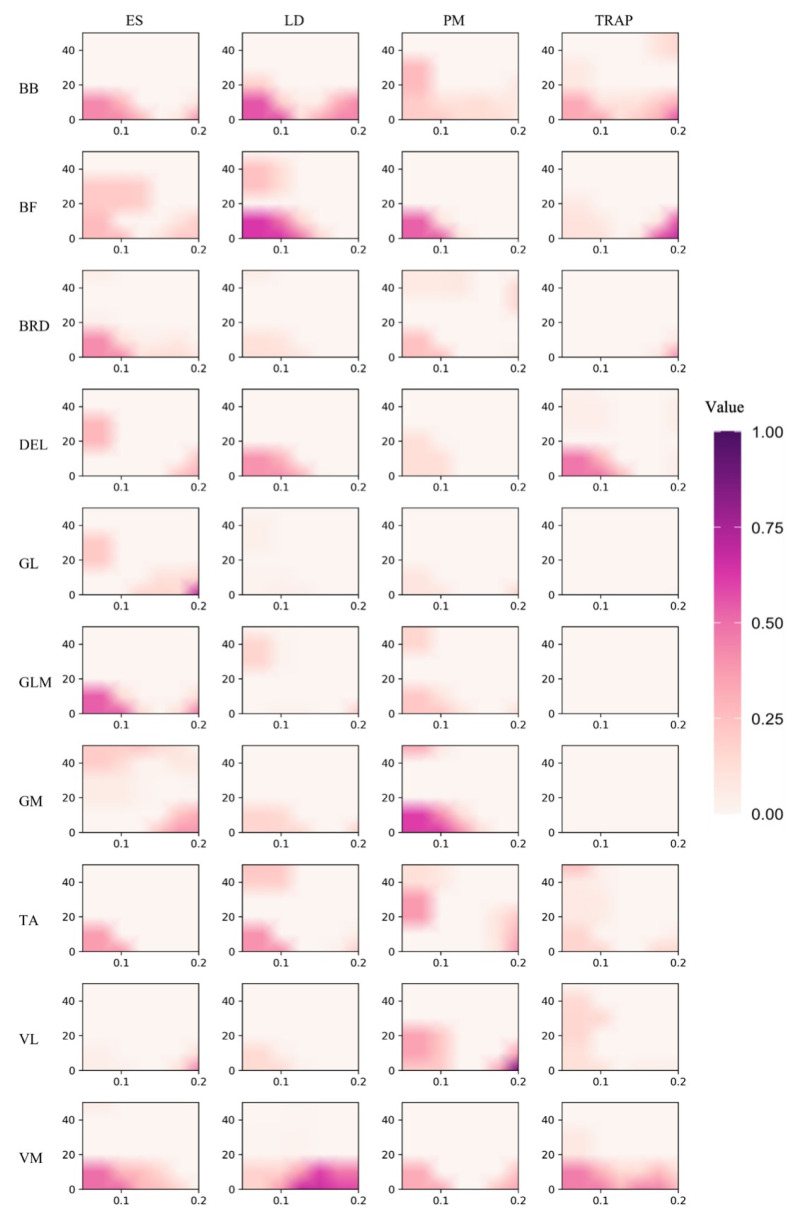
Intermuscular coherence heatmap for INT. Each panel shows the coherence between one muscle pair during the forehand stroke in the international-level group (INT). The column labels indicate trunk/upper-body reference muscles, and the row labels indicate paired limb muscles. The *x*-axis represents time, and the *y*-axis represents frequency in Hz. Color intensity represents coherence strength, with values ranging from 0 to 1; darker colors indicate stronger coherence. This figure shows the time–frequency distribution of intermuscular coupling across selected muscle pairs in INT players.

**Figure 6:**
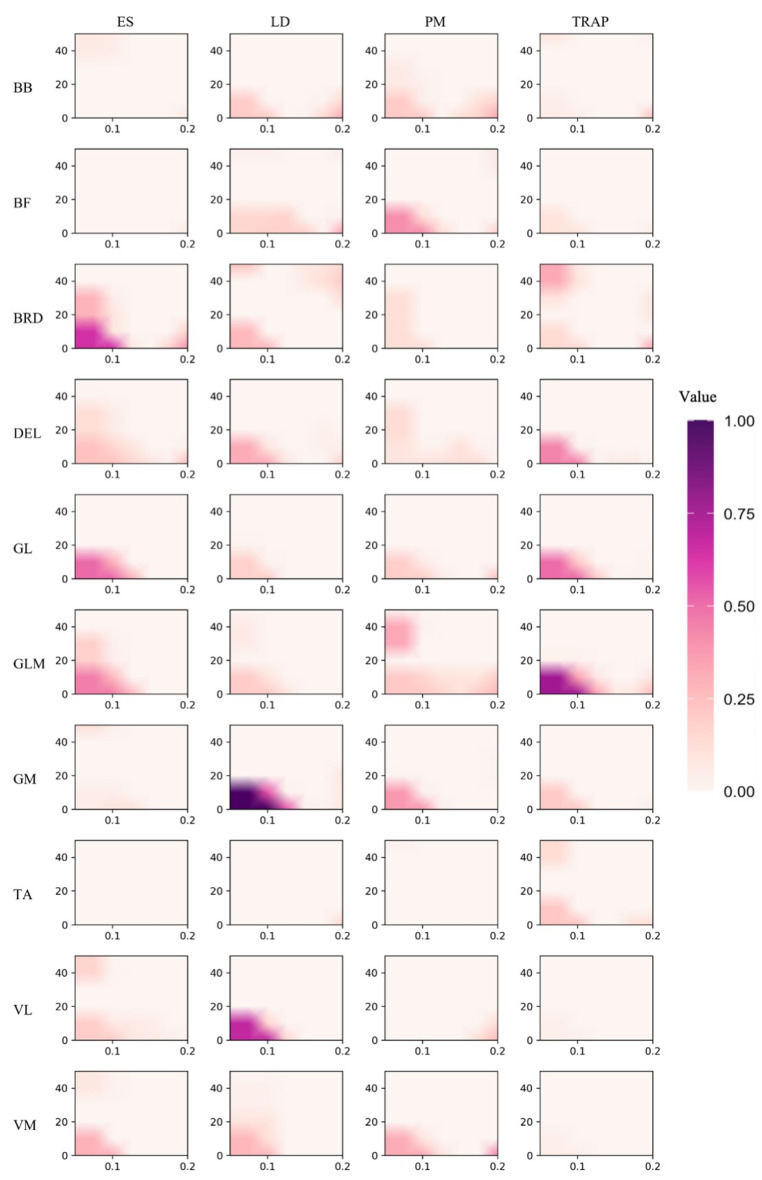
Intermuscular coherence heatmaps for the national-level group (NAT). Each panel shows the time–frequency coherence distribution between one selected muscle pair during the forehand stroke in national-level players. The *x*-axis represents time within the analysis window, and the *y*-axis represents frequency in Hz. Color intensity represents coherence strength, with values ranging from 0 to 1; higher values indicate stronger intermuscular coherence. The heatmaps show the distribution of trunk–limb intermuscular coupling across time and frequency in the NAT group.

**Figure 7:**
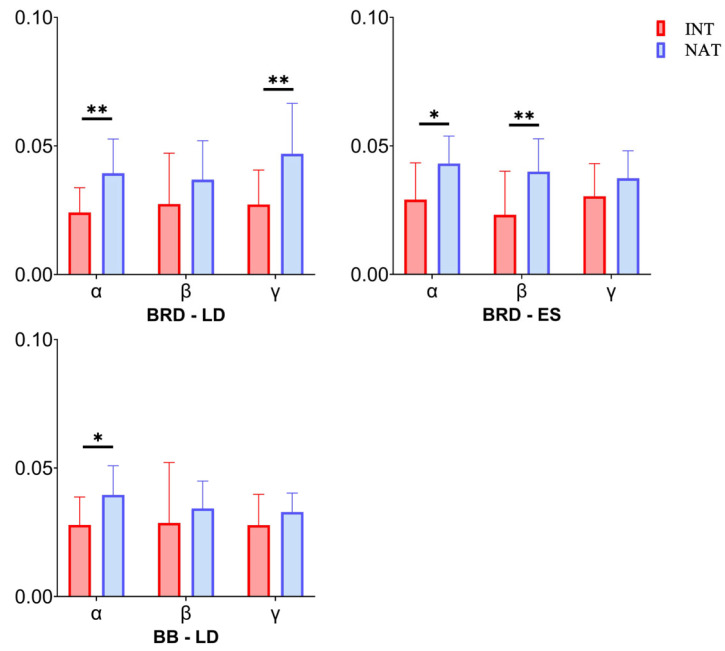
Comparison of intermuscular coherence for upper limb–trunk muscle pairs between the international-level group (INT) and national-level group (NAT). Az represents the area of significant coherence within the specified frequency band and was used to quantify coherence strength for each muscle pair. Bars show group mean values, and error bars represent standard deviations. Comparisons were performed for the α (8–15 Hz), β (15–30 Hz), and γ (30–50 Hz) frequency bands. Significance markers indicate between-group differences based on Benjamini–Hochberg FDR-adjusted *p* values: * FDR-adjusted *p* < 0.05; ** FDR-adjusted *p* < 0.01.

(Figure 5 and Figure 6) Coherence strength was quantified using the Az metric. For upper limb–trunk muscle pairs (Figure 7), the Az values of BRD–LD were lower in the INT group than in the NAT group in both the α and γ bands (*p* = 0.018 and *p* = 0.009, respectively). The Az values of BRD–ES were also lower in INT than in NAT in the α and β bands (both *p* = 0.012). In addition, the Az value of BB–LD in the α band was lower in INT than in NAT (*p* = 0.004).

**Figure 8:**
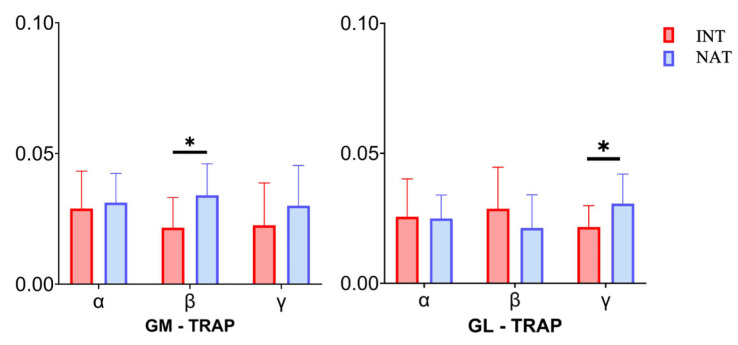
Comparison of intermuscular coherence for lower limb–trunk muscle pairs between the international-level group (INT) and national-level group (NAT). Az represents the area of significant coherence within the specified frequency band and was used to quantify coherence strength for each muscle pair. Bars show group mean values, and error bars represent standard deviations. Comparisons were performed for the α (8–15 Hz), β (15–30 Hz), and γ (30–50 Hz) frequency bands. Significance markers indicate between-group differences based on Benjamini–Hochberg FDR-adjusted *p* values: * FDR-adjusted *p* < 0.05.

For lower limb–trunk muscle pairs (Figure 8), the Az value of GM–TRAP in the β band was lower in INT than in NAT (*p* = 0.017), and the Az value of GL–TRAP in the γ band was lower in INT than in NAT (*p* = 0.038).

## 4. Discussion

This study compared neuromuscular control during the squash forehand stroke between international-level and national-level players using muscle synergy and intermuscular coherence analyses. The two groups did not differ significantly in the total number of extracted muscle synergies, but differed in the muscle weighting distribution and activation duration of the matched synergy patterns, as well as in intermuscular coherence. These findings suggest that performance-level differences in the squash forehand may be associated with differences in how comparable neuromuscular modules are organized, timed, and coupled across selected trunk–limb muscle pairs. However, because racket speed, ball velocity, stroke accuracy, joint kinetics, and other direct performance outcomes were not analyzed in this study, the present findings should be interpreted as evidence of differences in neuromuscular organization rather than direct evidence of greater stroke efficiency or superior performance.

### 4.1. Comparison of the Number of Synergies

The present study found no significant difference in the total number of extracted muscle synergies between international-level and national-level squash players. Under the VAF > 0.90 criterion, both groups required approximately five synergies on average to reconstruct the EMG data. This suggests that the overall dimensionality of the neuromuscular control scheme required for the forehand stroke was broadly similar between the two groups.

It should be noted that the total number of extracted synergies and the number of synergy patterns used for subsequent between-group comparisons are not identical. The synergy number reported in Table 2 reflects the number of modules required to reconstruct each participant’s EMG signals according to the VAF criterion. In contrast, the analyses of muscle weightings and activation durations were based on the group-level clustering and matching procedure [27]. After clustering and cross-group matching, four common and consistently matched synergy patterns were identified across the INT and NAT groups and were labeled SYN1–SYN4. Therefore, Table 3 and Table 4 focused on these four matched synergies because they represented the comparable synergy patterns suitable for between-group statistical analysis.

The additional extracted synergy components contributed to EMG reconstruction in some participants but were not consistently matched across groups according to the predefined matching criterion. Therefore, they were not included in the between-group comparisons of muscle weighting coefficients and activation duration. This distinction explains why approximately five synergies were extracted on average, whereas the pattern-level analyses were presented for SYN1–SYN4.

Accordingly, the absence of a significant between-group difference in the total number of synergies should not be interpreted as evidence that the two groups used identical neuromuscular strategies. Rather, it indicates that the overall dimensionality of EMG reconstruction was similar between groups, while the subsequent analyses of SYN1–SYN4 showed that the common matched synergy patterns differed in their internal muscle weighting distribution and temporal recruitment. Similar findings have been reported in archery, rowing, and tennis forehand movements [28], where athletes of different skill levels showed similar numbers of synergies. This indicates that synergy number may be more strongly constrained by task demands than by expertise itself. Therefore, skill-related differences may appear more clearly in the internal organization and temporal deployment of comparable synergy patterns than in the total number of extracted synergies.

### 4.2. Differences in Muscle Weightings

Although the total number of extracted synergies was similar, the two groups differed in the weighting coefficients of several muscles within the matched synergy patterns, including GL, DEL, PM, BB, BRD, ES, and GM. These differences suggest that international-level and national-level players may rely on different distributions of muscle contribution within comparable neuromuscular modules.

Specifically, in SYN1, the weighting of GL was lower in the INT group than in the NAT group. In SYN2, the weightings of DEL and PM were higher in INT than in NAT. In SYN3, the weighting of BB was lower in INT than in NAT, whereas the weightings of BRD and ES were higher in INT than in NAT; additionally, the weighting of PM was lower in INT than in NAT. In SYN4, the weighting of GM was higher in INT than in NAT. These results indicate that the between-group differences were not limited to a single anatomical region, but involved muscles associated with lower-limb support, trunk control, shoulder movement, and forearm involvement.

From a biomechanical perspective, the squash forehand requires coordinated force transfer from the lower limbs and trunk to the shoulder, arm, and racket. Therefore, differences in muscle weighting distribution may reflect differences in how players organize contributions from different regions of the kinetic chain. The relatively greater weighting of some proximal and trunk-related muscles in INT players may indicate a different organization of trunk–shoulder and lower-limb support within the matched synergy patterns. Conversely, greater reliance on selected distal or smaller muscles in NAT players may reflect a different distribution of control demands during the stroke.

However, muscle weighting coefficients should not be interpreted as direct measures of muscle force, isolated muscle activation amplitude, or mechanical power. A muscle weighting coefficient represents the relative contribution of a muscle within a multi-muscle synergy module. Thus, the present results indicate altered coordination organization rather than simply stronger muscle output. Without direct performance indicators such as racket speed, ball velocity, or stroke accuracy, the present findings cannot determine whether the observed muscle weighting patterns produced superior stroke outcomes.

This interpretation is consistent with evidence from tennis, badminton, and archery. Elite tennis players have been reported to coordinate large muscle groups more effectively [29], and higher-level tennis players show more focused activation of prime movers [30]. Advanced badminton players also demonstrate trunk- and forearm-related synergy characteristics [31], while elite archers rely on coordinated trunk stabilization [32]. In contrast, similar synergy morphology between elite and non-elite badminton players suggests that expertise-related differences may appear more in muscle allocation and timing than in gross synergy structure [33]. The present findings therefore suggest that competitive level in squash may be associated with differences in the internal organization of matched synergy patterns, rather than a fundamentally different number of neuromuscular modules.

### 4.3. Differences in Synergy Activation Duration

International-level players showed shorter activation durations in SYN1 and SYN2, but a longer activation duration in SYN3. No significant between-group difference was observed for SYN4. These findings suggest that the timing of synergy recruitment may be an important feature distinguishing the two groups.

The shorter activation durations of SYN1 and SYN2 in the INT group may indicate a more temporally concentrated recruitment of these matched modules during specific portions of the forehand stroke. During preparation and early swing, prolonged activation may reflect a broader or less selective recruitment window, whereas shorter activation may indicate a more time-specific recruitment pattern. In contrast, the longer activation duration of SYN3 in the INT group may suggest more sustained recruitment of this module during the analyzed stroke window, potentially related to control demands around ball contact and follow-through. Overall, international-level players appear to show a different phase-specific temporal organization, characterized by shorter activation of SYN1 and SYN2 and longer activation of SYN3 during the forehand stroke window.

This interpretation is consistent with tennis and badminton findings showing that skilled athletes may exhibit more task-specific temporal organization of multi-muscle coordination [34]. Similar late-phase co-activation has also been reported in wheelchair tennis [35], and elite tennis players have been shown to use follow-through activation strategies that may contribute to joint stabilization [36]. However, the functional meaning of the activation duration differences in the present study should be interpreted cautiously because no direct kinetic or performance measures were collected.

Activation duration was defined as the time interval during which the synergy activation coefficient exceeded 0.30. Therefore, the absolute duration values may be affected by the selected threshold, smoothing method, time normalization, and the defined analysis window. These results should be interpreted as relative indicators of temporal organization within the present analytical framework rather than exact durations of neural command. In addition, because racket speed, ball velocity, and stroke accuracy were not analyzed, the observed timing differences cannot be interpreted as direct evidence of greater movement efficiency. Instead, they indicate that the two groups differed in how the matched synergy modules were temporally recruited during the standardized forehand task.

### 4.4. Differences in Time–Frequency Intermuscular Coherence

International-level players showed lower IMC in selected α, β, and γ frequency bands for upper limb–trunk and lower limb–trunk muscle pairs. This indicates that the groups differed not only in muscle synergy organization, but also in intermuscular coupling. IMC is commonly used to assess common oscillatory input to muscles, and coherence in different frequency bands may reflect different components of neuromuscular control [37,38]. Coherence across coactivated muscles can also reflect functional coordination and shared neural input [39].

One possible interpretation is that international-level players relied less on generalized common coupling across selected trunk–limb muscle pairs. During the squash forehand, different body regions have different mechanical roles: the lower limbs provide support and propulsion, the trunk contributes rotation and stabilization, the shoulder–arm complex accelerates the racket, and the forearm supports fine control near impact. Lower coherence in selected muscle pairs may therefore reflect more differentiated coupling among these regions. However, because direct performance outcomes were not measured, this interpretation should be regarded as a possible explanation rather than direct evidence of greater neuromuscular efficiency.

The physiological meaning of lower IMC should also be interpreted cautiously. Higher coherence is not always worse, and lower coherence is not always better. IMC depends on task demands, contraction intensity, movement phase, muscle pair, frequency band, and signal-processing choices. Thus, the reduced coherence in international-level players should be interpreted as evidence of altered intermuscular coupling, not definitive proof of superior neural control. In addition, surface EMG-based coherence cannot directly identify the exact source of common drive. Therefore, the IMC findings should be viewed as indirect evidence of differences in neuromuscular coordination.

### 4.5. Integrated Interpretation

Overall, the present findings suggest that international-level squash players did not perform the forehand stroke by using a significantly different total number of extracted muscle synergies. Instead, both groups required approximately five synergies on average to reconstruct the EMG data, and four common synergy patterns were consistently matched between groups and analyzed as SYN1–SYN4. Within these matched synergy patterns, the INT and NAT groups differed in muscle weighting distribution and activation duration. In addition, the two groups showed different intermuscular coherence patterns in selected trunk–limb muscle pairs.

Together, these findings indicate that competitive level may be associated with differences in neuromuscular organization during the squash forehand. The weighting results indicate different muscle contribution patterns within matched synergy modules; the activation duration results indicate different temporal recruitment of these modules; and the IMC results suggest altered trunk–limb intermuscular coupling. These results provide complementary information about neuromuscular coordination during the forehand stroke.

However, the findings should not be interpreted as direct evidence that international-level players produced faster, more accurate, or mechanically more efficient strokes. The present study did not measure racket speed, ball velocity, stroke accuracy, joint kinetics, or energy transfer. Therefore, although the observed neuromuscular differences may be relevant to performance-level differences, their direct relationship with actual stroke performance remains to be tested. From a training perspective, these findings suggest that coaches and practitioners may consider assessing whole-body neuromuscular coordination, especially trunk–shoulder coordination, proximal force transmission, and controlled follow-through. However, future studies are needed to determine whether these neuromuscular patterns are directly associated with measurable improvements in squash performance.

### 4.6. Limitations and Future Directions

Several limitations should be noted. First, this study used a cross-sectional design, so it cannot determine whether the observed differences were caused by long-term elite training or reflected pre-existing characteristics. Second, the sample size was limited, especially in the international-level group, which may affect statistical power and generalizability. Third, all participants were male and right-handed, so the findings may not apply to female players, left-handed players, or athletes with different technical styles.

Fourth, the forehand stroke was tested under standardized conditions using a ball-feeding machine and a predefined target area. This improved experimental control but may not fully reproduce the perceptual and tactical demands of real match play. Fifth, this study did not directly measure racket speed, ball velocity, stroke accuracy, joint kinetics, or kinetic-chain energy transfer. Therefore, the observed differences in muscle weighting distribution, synergy activation duration, and intermuscular coherence should be interpreted as differences in neuromuscular organization rather than direct evidence of greater stroke efficiency or superior performance outcomes.

Sixth, surface EMG has inherent limitations, including possible cross-talk, sensitivity to electrode placement, and difficulty detecting deep muscles. The selected muscle set also did not include all muscles involved in the squash forehand, such as deep trunk stabilizers or smaller shoulder stabilizers. Finally, muscle synergy and IMC outcomes are influenced by methodological choices, including preprocessing, normalization, VAF threshold, factorization algorithm, synergy matching procedure, activation-duration threshold, window length, smoothing, frequency-band definitions, and selected muscle pairs. Therefore, the findings should be interpreted as method-dependent indicators of neuromuscular organization rather than direct measurements of neural commands.

Future studies should combine synergy and coherence analyses with direct biomechanical and performance measures, including racket speed, ball velocity, stroke accuracy, joint kinetics, and kinetic-chain energy transfer. Longitudinal training interventions are also needed to determine whether specific neuromuscular organization patterns can be modified through training and whether such changes are associated with measurable improvements in squash forehand performance.

## 5. Conclusions

This study compared muscle synergy and intermuscular coherence characteristics during the forehand stroke between international-level and national-level squash players. The two groups showed no significant difference in synergy number, suggesting that the basic modular control structure of the squash forehand is relatively stable across performance levels. However, international-level players demonstrated more refined neuromuscular organization, characterized by greater reliance on proximal major muscles, more phase-specific synergy activation timing, and lower intermuscular coherence in selected trunk–limb muscle pairs. These findings indicate that higher-level squash forehand performance may be associated not with using more muscle synergies, but with more efficient tuning of existing neuromuscular modules and more flexible intermuscular coordination. From a training perspective, emphasis should be placed on trunk–shoulder coordination, proximal force transmission, controlled follow-through, and variable stroke practice to improve kinetic chain efficiency and neuromuscular control.

## Figures and Tables

**Table 1:** Participant characteristics (mean ± SD).

Group	Age (Years)	Height (cm)	Body Mass (kg)	Dominant Side	Injury History
INT	27.6 ± 2.8	185.0 ± 4.0	74.7 ± 7.4	Right-handed	None
NAT	22.5 ± 4.1	179.0 ± 6.0	72.5 ± 7.7	Right-handed	None

**Table 2:** Number of muscle synergies in the INT and NAT groups.

INT		NAT		*p*
Mean	sd	Mean	sd	
5.00	0.60	5.08	0.67	0.8469

**Table 3:** Muscle weightings for SYN1–SYN4 in INT and NAT players. *p* values are FDR-adjusted within each synergy across the 14 muscle comparisons.

Muscle	SYN	INT		NAT		*p*	SYN	INT		NAT		*p*
		Mean	sd	Mean	sd			Mean	sd	Mean	sd	
BB	SYN1	0.32	0.29	0.16	0.15	0.1432	SYN2	0.12	0.20	0.16	0.22	0.4789
BF		0.22	0.31	0.24	0.21	0.5573		0.06	0.13	0.10	0.15	0.5962
BRD		0.31	0.24	0.35	0.37	0.8094		0.22	0.19	0.21	0.19	0.9295
DEL		0.13	0.19	0.10	0.15	0.6539		0.33	0.28	0.06	0.12	0.0204
ES		0.13	0.23	0.12	0.19	0.9177		0.22	0.23	0.23	0.27	>0.9999
GL		0.03	0.04	0.14	0.16	0.0159		0.08	0.10	0.12	0.21	0.536
GLM		0.23	0.28	0.14	0.22	0.5573		0.11	0.22	0.28	0.24	0.0556
GM		0.06	0.18	0.10	0.18	0.3494		0.16	0.22	0.19	0.18	0.5962
LD		0.08	0.07	0.08	0.13	0.4262		0.23	0.19	0.13	0.12	0.1851
PM		0.04	0.09	0.07	0.13	0.9177		0.32	0.24	0.03	0.05	0.0204
TA		0.11	0.11	0.20	0.35	0.5573		0.17	0.22	0.38	0.36	0.1821
TRAP		0.34	0.31	0.24	0.36	0.3867		0.15	0.17	0.17	0.25	>0.9999
VL		0.07	0.11	0.03	0.06	0.4679		0.15	0.18	0.08	0.15	0.4252
VM		0.05	0.09	0.07	0.09	0.9177		0.10	0.20	0.11	0.15	0.2463
BB	SYN3	0.03	0.07	0.18	0.21	0.0381	SYN4	0.05	0.09	0.17	0.24	0.0879
BF		0.06	0.13	0.07	0.08	0.4561		0.04	0.07	0.06	0.04	0.2703
BRD		0.51	0.39	0.17	0.16	0.0351		0.37	0.24	0.27	0.21	0.2426
DEL		0.09	0.13	0.10	0.16	0.7664		0.07	0.12	0.15	0.17	0.133
ES		0.21	0.29	0.06	0.10	0.0465		0.22	0.29	0.23	0.21	0.6994
GL		0.13	0.22	0.08	0.14	0.6027		0.22	0.17	0.18	0.21	0.4009
GLM		0.21	0.20	0.24	0.17	0.7591		0.07	0.09	0.07	0.10	0.6994
GM		0.09	0.15	0.20	0.24	0.261		0.27	0.23	0.11	0.21	0.04
LD		0.14	0.16	0.26	0.23	0.1308		0.16	0.19	0.18	0.23	0.9487
PM		0.11	0.08	0.45	0.24	0.0008		0.00	0.01	0.03	0.07	0.3
TA		0.04	0.05	0.12	0.17	0.5516		0.29	0.29	0.18	0.26	0.3653
TRAP		0.02	0.04	0.05	0.11	0.9408		0.12	0.16	0.22	0.28	0.5619
VL		0.19	0.27	0.22	0.22	0.3312		0.18	0.23	0.19	0.23	0.6994
VM		0.20	0.26	0.19	0.25	0.9408		0.20	0.25	0.24	0.28	0.6994

Note: *p* values are Benjamini–Hochberg FDR-adjusted *p* values. FDR correction was applied separately within each synergy across the 14 muscle comparisons.

**Table 4:** Activation duration of SYN1–SYN4 in the INT and NAT groups. *p* values are FDR-adjusted across the four synergy comparisons.

SYN	INT		NAT		*p*
	Mean	sd	Mean	sd	
SYN1	34.5	7.122	52.09	11.7	0.0006
SYN2	17.14	5.242	38.64	11.18	0.0002
SYN3	54.78	15.07	24.55	8.43	<0.0001
SYN4	34.45	5.646	36.55	13.97	0.6528

Note: *p* values are Benjamini–Hochberg FDR-adjusted *p* values. FDR correction was applied across the four synergy activation–duration comparisons.

## Data Availability

The original contributions presented in this study are included in the article. Further inquiries can be directed to the corresponding author.
